# FedECPA: An Efficient Countermeasure Against Scaling-Based Model Poisoning Attacks in Blockchain-Based Federated Learning †

**DOI:** 10.3390/s25206343

**Published:** 2025-10-14

**Authors:** Rukayat Olapojoye, Tara Salman, Mohamed Baza, Ali Alshehri

**Affiliations:** 1 Department of Computer Science, Texas Tech University, Lubbock, TX 79409, USA; rolapojo@ttu.edu (R.O.); tsalman@ttu.edu (T.S.); 2 Department of Computer Science, College of Charleston, Charleston, SC 29424, USA; 3 Department of Computer Science, University of Tabuk, Tabuk 71491, Saudi Arabia; a.alshehri@ut.edu.sa

**Keywords:** federated learning, blockchain, smart contract, scaling-based model poisoning attack, FedECPA, security, IoT

## Abstract

Artificial intelligence (AI) and machine learning (ML) have become integral to various applications, leveraging vast amounts of heterogeneous, globally distributed Internet of Things (IoT) data to identify patterns and build accurate ML models for predictive tasks. Federated learning (FL) is a distributed ML technique developed to learn from such distributed data while ensuring privacy. Nevertheless, traditional FL requires a central server for aggregation, which can be a central point of failure and raises trust issues. Blockchain-based federated learning (BFL) has emerged as an FL extension that provides guaranteed decentralization alongside other security assurances. However, due to the inherent openness of blockchain, BFL comes with several vulnerabilities that remain unexplored in literature, e.g., a higher possibility of model poisoning attacks. This paper investigates how scaling-based model poisoning attacks are made easier in BFL systems and their effects on model performance. Subsequently, it proposes FedECPA-an extension of FedAvg aggregation algorithm with Efficient Countermeasure against scaling-based model Poisoning Attacks in BFL. FedECPA filters out clients with outlier weights and protects the model against these attacks. Several experiments are conducted with different attack scenarios and settings. We further compared our results to a frequently used defense mechanism, Multikrum. Results show the effectiveness of our defense mechanism in protecting BFL from these attacks. On the MNIST dataset, it maintains an overall accuracy of 98% and 89% and outperforms our baseline with 4% and 38% in both IID and non-IID settings, respectively. Similar results were achieved with the CIFAR-10 dataset.

## 1. Introduction

The Internet of Things (IoT) is an integrated system of billions of smart devices, such as smartphones and sensor networks, that communicate over local and global networks to make certain decisions that currently affect our daily lives. These smart devices are a source of massively distributed data collected daily from various mobile and remote applications. Traditionally, such data are sent to a single machine or a data center for training using conventional ML techniques. Such data transfer risks privacy leakage from untrusted third parties, preventing data owners from uploading data to the central server [1]. Furthermore, raw data transfer entails substantial communication overhead, particularly given the prevalent use of cloud-based central servers.

*Federated learning (FL)* is a new ML approach that offers collaborative learning from massively distributed protected data that does not leave IoT devices [2]. Aside from ensuring data privacy, FL also facilitates access to heterogeneous IoT data, which can improve the overall performance of ML models. In an FL system, each party trains a local model using its own IoT data and exchanges only the model parameters with a centralized server for aggregation. The server aggregates the clients’ local models to generate a new global model to be shared with the clients and used in subsequent local iterations for training [3]. Therefore, the server is a central player that has to be trusted and potentially represents a single point of failure [4]. In addition, FL does not guarantee local model security as some untrustworthy FL servers can manipulate model parameters submitted by clients [5]. The integrity of local and global models cannot be verified in FL, which indicates a possibility of fake local model submissions by malicious clients and global models generated by the server [6]. In peer-to-peer FL, each client generates a local model similar to the conventional FL. Additionally, they communicate directly with other clients rather than the central server and aggregate the local updates from a group of random neighboring clients [7]. Although this scheme eliminates the need for a central server, the issue of trust among the clients and the security of their local models remains unsolved.

Fortunately, the emerging blockchain technology resolves these aforementioned issues [8]. The central server can be eliminated and replaced with the blockchain network. In this way, local models can be stored, exchanged, and merged without a centralized server [3]. In addition, the blockchain system’s transparency, traceability, and immutability eliminate local model security concerns. Local models submitted to the network are tamper-proof unless the system security is broken [9]. Such a system also ensures non-fake local and global models by allowing nodes to verify submitted and generated models [10]. Consequently, several works have proposed blockchain-based federated learning (BFL) systems that resolve the issues with traditional FL systems [11,12,13,14,15,16,17] and IoT [18,19,20,21]. These approaches offer valuable insights, yet they each suffer from critical shortcomings that limit their effectiveness in practice.

For instance, Krum [22] and Shield-FL [23] perform well against isolated outliers but fail when a majority of clients are malicious or when poisoned updates are close to the benign aggregation space. Blade-FL [15] provides strong integrity and privacy guarantees but lacks mechanisms for detecting malicious clients, making them vulnerable to stealthy model poisoning. Cryptographic methods like ADFL [24] ensure verifiability but introduce substantial computational overhead and remain weak against stealthy adversaries. Similarly, prediction-based detection methods like FLDetector [25] can identify sudden deviations but are easily bypassed by consistent poisoning strategies. These limitations highlight a pressing need for a defense that not only leverages the security and transparency of blockchain but also incorporates robust malicious client detection during aggregation.

Moreover, the vulnerabilities of BFL have not been extensively researched. *Due to the openness of the blockchain network, all participants have access to critical information in the BFL*. Malicious clients can leverage this knowledge to launch attacks that are more difficult to launch on traditional FL. For example, some model poisoning attacks require knowing the number of participating clients, which is only known to the server in a conventional FL, but can be inferred from the number of blocks and transactions by any participant in the BFL system. Consequently, BFL makes it feasible for adversaries to launch attacks on the FL system, potentially leading to prediction errors in critical IoT applications such as healthcare, intrusion detection, and autonomous vehicles. For instance, if hospitals employ a poisoned global BFL model for mortality prediction, it could lead to patient fatalities in the event of catastrophic incorrect predictions.

Thus, our proposed scheme, FedECPA, directly addresses these gaps. FedECPA employs the Interquartile Range (IQR) to filter out anomalous client models in each training round. Since IQR is a robust statistical measure that captures the spread of the central 50% of the data while minimizing the influence of extreme values, it is particularly effective for detecting outliers in model weights. By identifying and excluding weights that fall outside the lower and upper IQR limits, FedECPA ensures that only reliable client updates contribute to the global model aggregation. A preliminary result of this work was accepted in [26]. The key differences in the current version include the exploration of additional attack settings and scenarios, the proposition of a practical defense mechanism, evaluations of the defense mechanism in both IID and non-IID settings, a comparative analysis with a baseline method, Multikrum, and evaluation of the blockchain implementation. The contributions of this work are summarized as follows:

1. A practical scaling-based model poisoning attack on a BFL system is introduced. Specifically, we simulate an FL task with non-malicious and malicious clients for different attack strategies and settings, such that malicious clients use model weights on the blockchain to perform the attack.
2. A novel defense, named FedECPA, an extension to FedAvg aggregation algorithm with Efficient Countermeasure against scaling-based model Poisoning Attacks, is proposed. The main idea of this defense is to employ the interquartile range (IQR) rule as a statistical method to filter out outlier model weights submitted by malicious clients. Specifically, model weights that fall outside of a lower and upper limit (based on the 25th and 75th percentiles of the participating clients’ model weights) are identified and excluded from aggregation.
3. A comprehensive security analysis of FedECPA, demonstrating its Byzantine tolerance to mitigate scaling attacks effectively.
4. Extensive experimental and comparative analyses are conducted in both IID and non-IID settings to demonstrate the impact of the model poisoning attack on BFL and the efficacy of the proposed defense mechanism. Results show that FedECPA, in most cases, outperforms Multikrum [27]—a traditionally robust FL aggregation algorithm that mitigates model poisoning attacks in most FL settings.

The rest of the paper is organized as follows. Section 2 discusses the related works. Section 3 presents background on blockchain systems, FL, and BFL. Section 4 highlights the system model, threat model, and security goals. Section 5 discusses the practical model poisoning attack in BFL, the proposed defense mechanism, BFL implementation, and the complexity analysis of FedECPA. Section 6 explains in detail the experimental analysis, including the implementation setup, the model and data settings, the performance evaluation of the BFL, and the proposed defense mechanism. It also presents the security analysis of FedECPA. Finally, Section 7 concludes the paper and presents some future work.

## 2. Related Work

Recently, several research works have focused on integrating blockchain with FL. In the works of [11,12,13,28], smart contracts were used for the automatic execution of FL. Clients participate in the execution of a smart contract by sending their local models for aggregation, making it accessible to everyone in the system. In [29], a mobile crowdsensing learning framework was proposed, consisting of a requester, blockchain consortium, edge servers (FL clients), and mobile devices (data source). After the requester publishes a task to the blockchain network, mobile devices collect data and send it to the edge server (FL clients) for training. However, the authors did not consider the quality of the models submitted by the edge servers to the blockchain network. In Blade-FL [15], each client also plays the roles of a trainer and a miner. Aside from managing the blockchain network with internal clients to lessen privacy leakage, Blade-FL also integrated the proof of work (PoW) consensus for model updates to preserve the system’s security. However, malicious client detection was not taken into consideration. Our work extensively studies the impact of falsified clients’ local model weights on the global model and the overall BFL system. Additionally, we consider several settings that malicious clients could employ and new threat models brought by using blockchain to run the BFL system.

In terms of mitigating model poisoning attacks, several other research works have been proposed in [24,27,30,31] as summarized in Table 1.

Krum [27], as proposed and implemented in [22], is an aggregation method that selects the local model most similar to the others among *m* local models to serve as the global model. This approach helps limit the potential impact of a malicious model, since even if the selected model originates from an attacker, its closeness to other models likely from benign clients reduces harmful deviations. However, a scenario in which a more significant percentage of the clients are malicious was not considered, as the global model selection will be misguided. In Shield-FL [23], the clients’ local weights and the global weights are represented as vectors with the local weights being multi-dimensional. The outliers were identified by computing the cosine similarity between local and aggregated weights. This approach will be ineffective if an adversary computes a poisoned model weight close to the aggregated weights.

A defense mechanism for horizontal FL was also proposed in ADFL [24], which uses a proof generation method for clients to generate proofs based on a garbled aggregation model to verify whether they are adversaries. In FLDetector [25], the server predicts a client’s model update in each iteration based on its model updates from previous iterations. A consistency score is awarded to each client, and thus, a malicious client is identified when the model update from the client is inconsistent with the predicted model update. However, this work fails to consider malicious clients that launch an attack in every learning iteration, thereby submitting consistent yet erroneous local model weights. On the contrary, our work detects the malicious clients’ model weights at every learning iteration using the IQR technique to identify the variation in the clients’ model weights. Consequently, the outlier weights submitted by the malicious clients are excluded before aggregation.

## 3. Background

This section provides a background on blockchain and FL needed to understand this work.

### 3.1. Blockchain and Smart Contract

Blockchain is a secured, shared, and distributed ledger of transactions across an entire network of nodes, making it easier to record and track resources without a centralized authority [32]. All nodes in the system agree on the transactions to be saved and their order [33]. The system can be public or private. A public blockchain system allows the participating nodes to join and exit the network without permission. Also, they are allowed to participate in the consensus process and access the public ledger. On the contrary, in a private blockchain system, the participating nodes are supervised, so only authorized nodes can join that network and access the shared ledger [1]. The architecture of a blockchain system consists of a network of globally distributed nodes (otherwise named miners) and the blockchain ledger. The nodes are the users or highly configured devices responsible for the transactions carried out in the system. Typically, they verify and validate blocks of transactions using a consensus protocol [34]. The ledger consists of chained blocks, each composed of several transactions representing the system’s main interactions.

Smart contracts are self-executing programs deployed on blockchain platforms that automatically enforce agreements among multiple parties using software code and computational infrastructure [33,35]. The architecture of a smart contract consists of several integrated layers that work together to ensure secure and reliable execution. The contract code layer contains the immutable program logic that defines the rules, conditions, and actions of the contract. The ledger layer records all transactions triggered by the contract on the blockchain, providing transparency, auditability, and a permanent record of all interactions. The consensus layer ensures that all contract executions are validated according to the blockchain’s underlying protocol, guaranteeing agreement among all nodes. The execution layer handles the computation of contract logic when invoked by participants, including reading input data, performing calculations, updating the ledger, and triggering subsequent actions. Finally, the interface layer provides APIs or function calls that enable participants to interact with the smart contract, ensuring accessibility and allowing authorized users to submit data, query results, or invoke operations.

It is important to note that each smart contract execution incurs a gas cost, which represents the computational and storage resources consumed on the blockchain. While manageable at small scales, these costs can accumulate in large-scale FL deployments where frequent model updates and verifications may be required. This introduces a potential scalability bottleneck, as excessive gas usage can increase latency and operational costs. To address this, we optimized our implementation by storing model weights as 256-bit integers and representing them as flattened arrays, which enables efficient arithmetic operations in Solidity and reduces on-chain computation overhead. The detailed design and implementation of our smart contracts are provided in Section 5.

### 3.2. Federated Learning (FL)

FL is a distributed ML technique that allows devices to train data locally and upload local model updates to a central server. These local model updates can be in the form of model weights [12] or model parameters [16]. The central server runs a predefined aggregation algorithm to obtain a global model that will be used for training in further iterations. In FL literature, these local devices have been called clients, participants, workers, or simply devices, while the server can also be called an aggregator. For the rest of this paper, we will use clients to represent these local devices and the server to represent the central server [36]. There are generally three types of FL depending on how the data is partitioned across clients. In horizontal FL [37], clients share the same feature space but hold different samples, which is the most common setting in practice. In vertical FL [38], clients have different feature spaces but share the same set of users or identifiers. Finally, in federated transfer learning (FTL) [39], both the feature and sample spaces differ. Knowledge transfer techniques are used to enable collaboration. In this work, we adopt the horizontal FL. The process described in [1] is as follows:

- *Clients selection:* The server selects clients to participate in training at a given learning iteration as a first step. The selection can happen randomly, based on predefined protocols, or all clients can participate [40]. Several predefined selection protocols exist, including those based on effective participation and fairness [41] and greedy selection based on confidence bounds [42].
- *Local model training:* Next, selected clients download the global model parameters and train local models based on their data and the downloaded global model. In each iteration, clients compute
  (1) $$w_{i}^{t}=w_{i}^{t-1}-\eta \nabla f_{i}\left(W_{g}^{t-1};x_{i},y_{i}\right)$$
  where $w_{i}^{t}$ and $w_{i}^{t-1}$ are the local model weight of $client_{i}$ at iterations *t* and t−1, respectively [43]. $W_{g}^{t-1}$ is the global model at the learning iteration t−1, η is a learning rate (an ML hyper-parameter that controls how quickly an algorithm learns or updates the estimates of a parameter), $f_{i}$ is the local loss function for $client_{i}$, and $x_{i}$ and $y_{i}$ are local data with data size $d_{i}$ at client $client_{i}$.
- *Local model upload:* After the local training, clients upload local model updates to the server for aggregation.
- *Global model aggregation:* A new global model is calculated on the server by executing an aggregation algorithm that merges clients’ updates. These aggregation algorithms can calculate the mean, such as *FedAvg* [44], or add momentum to the calculated mean as in *FedAvgM* [45]. In this work, we adopt FedAvg, which is the conventional and most popular aggregation algorithm. In FedAvg, the global model is computed as
  (2) $$W_{g}^{t}=\sum_{i=1}^{n}\frac{d_{i}}{D}w_{i}^{t}$$
  where $w_{i}^{t}$ is the model update of each client $client_{i}$ at the learning iteration *t*, $d_{i}$ is the number of data samples for each client, and *D* is the total number of data samples from all participating clients.
- *Global model download:* After aggregation, the participating clients download the newly computed global model for the next training iteration.

### 3.3. Blockchain-Based Federated Learning (BFL)

As explained before, a primary motivation for integrating blockchain with FL is that blockchain has great potential to improve security for FL systems [36]. The shared, immutable ledger aggregates the global model and distributes global updates to learning clients for direct computation at devices. The participants of a BFL system are referred to as the *clients and nodes* [46]. The *clients* are FL clients who perform local training and generate local model updates. The *nodes* are blockchain nodes that verify the updates, execute consensus processes, perform aggregation to generate the global model, and write the new global model updates as a blockchain transaction. The clients then download the global model for the next training iteration. In a BFL, the local model uploading and global model updates are performed using smart contracts. In this case, the FL clients invoke the appropriate smart function to upload their local model updates and download the global model updates. However, the blockchain nodes invoke the proper functions to verify the local model updates and calculate the new global model.

## 4. System and Threat Model

In this section, we present the system model of BFL. We also discuss our threat model and security goals of this work.

### 4.1. System Model

**Figure 1:**
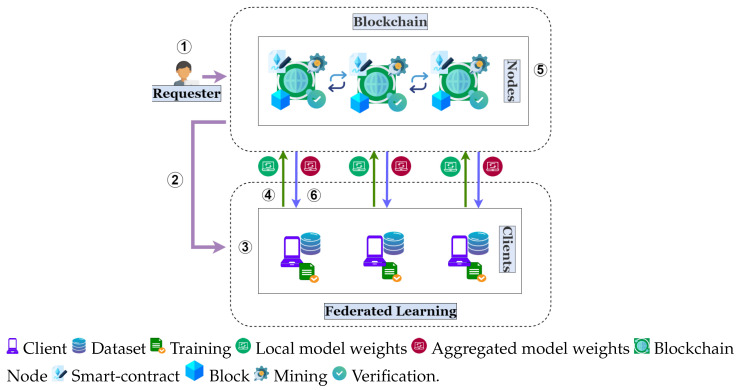
An illustration of BFL architecture. ➀ Global model request; ➁ request broadcast to FL clients; ➂ local model training with private datasets; ➃ local model weights sent to the blockchain nodes; ➄ local model weights update verification and aggregation; ➅ qggregated model weights sent back to clients.

As illustrated in Figure 1, our BFL system follows a horizontal FL setting, where all FL clients share the same feature space but hold data for different samples. The system consists of three main entities: a *Requester*, *FL Clients*, and *Blockchain Nodes*. The requester requests a global model to be computed. The FL clients train local models based on their private data and the current global model to generate their local model weights. Blockchain nodes are responsible for generating blocks and keeping track of the distributed ledger. They also hold all the smart contracts required for necessary operations and computations. The FL processes in a BFL system can be detailed in the following steps:

- *Step 1*: A requester requests a global model and publishes the task to the blockchain network.
- *Step 2*: The request is broadcast through the nodes in the network to FL clients.
- *Step 3*: Upon obtaining this request, FL clients train their local models based on their data and the current global model weights. This results in new local model weights.
- *Step 4*: After generating the local model weights, they are uploaded to the blockchain network for aggregation. This is done by submitting a transaction or calling the upload weights smart contract function.
- *Step 5*: Blockchain nodes collectively verify the local model weights from clients and record them on the blockchain ledger (in terms of transactions). They also aggregate these local model weights and generate the new global model.
- *Step 6*: Finally, the new global model is broadcast back to the participating clients for the next training iteration.

### 4.2. Threat Model

We assume that malicious client(s) are dishonest and that they attempt to compromise the global model. We consider a non-privacy-preserving blockchain (i.e., data and transactions are not encrypted). This consideration is done to ease the process of obtaining the model parameters and aggregating them by blockchain nodes, which is difficult to do in a privacy-preserving blockchain. Hence, our consideration of a non-privacy-preserving blockchain primarily aims to facilitate the implementation of the BFL process rather than to exploit any vulnerabilities. Privacy-preserving blockchains are well-documented in the literature [47], and our attack model and assumptions are generally viable with these blockchains, as will be elaborated later.

We also assume that less than 20% of the clients are malicious at a time or an iteration. Given the large number of clients generally involved in FL settings and the difficulty of compromising 20% of these clients at the same time or iteration, this assumption is reasonable. Further, we assume there is enough computation for the mining process in the blockchain system. The malicious client(s) require certain knowledge to compromise the global model. This knowledge can be classified as follows:

- *Private knowledge*: The malicious client(s) need(s) information about the number of participating FL clients (even if their model updates are encrypted). Although this information is highly restrictive server-side knowledge in a traditional FL, it will become non-private in BFL due to the open nature of blockchain. Since clients’ models are broadcast to all participating nodes, adversaries can exploit the visibility of these models to craft poisoning attacks. Moreover, once malicious clients’ models are recorded on-chain, they become irreversible, amplifying their negative effect on the global model. These malicious clients can infer this information from the number of blocks and transactions in the blockchain and leverage it to launch the model poisoning attack on the BFL system.
- *Public knowledge*: This includes the knowledge of the learning rate, the number of learning iterations, and the global model computed in each iteration. They are public parameters our malicious client(s) require. We assume that all client(s) participating in learning iterations have access to these parameters, and malicious clients can use them to launch model poisoning attacks.

### 4.3. Security Goals

Following the discussion of our system and threat model, this work aims to achieve the following security goals:

- *Unpoisoned global model*: This goal ensures that the global model is not poisoned, given our earlier assumption of less than 20% malicious clients. This means that we should only have non-malicious client(s) updates and eliminate poisoned updates. In other words, the malicious (poisoned) updates should not affect the global model.
- *Non-Fake model updates*: We aim to ensure that the local and global models are correctly calculated and guaranteed to be non-fake and authentic. In other words, we want to ensure that all generated global and local models are verified for correctness.
- *No single point of failure and no trusted third party*: Another goal is to ensure that the BFL is centralization-free by ensuring no single point (node or a party) of failure and no trusted third party is involved.

## 5. Proposed FedECPA Methodology

This section first discusses the model poisoning attack in BFL, the proposed defense mechanism for this attack, and the BFL implementation.

### 5.1. Model Poisoning Attacks

**Figure 2:**
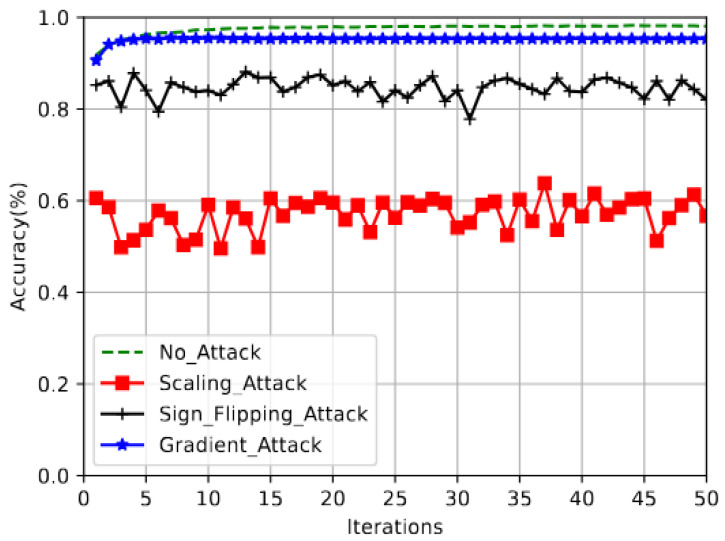
Illustration of various model poisoning attacks.

Model poisoning attacks are carried out by perturbing or tampering with the local model weights generated by malicious clients. Such tampering aims to poison the global model so that its performance degrades. Different strategies can be adopted in formulating such an attack. Some of the most common strategies include *Scaling attack* [48], where the malicious client simply multiplies its generated local model weights by a scale, *Sign flipping attack* [49], where the malicious client flips the signs of its model weights, and *Gradient attack* [50], where the malicious client maximizes its training loss rather than minimizing it. Figure 2 shows the effect of these strategies on the global model parameter. As can be seen, scaling attacks with a proper scale have the maximal effect on the global model. Thus, our primary focus in this paper is on scaling attacks, where the scale that we will define as the boosting factor follows the attack strategy in [48].

### 5.2. Attack Construction

By leveraging blockchain’s openness, malicious clients may query the current local weights to estimate the proper malicious (poisoned) weight, thereby maximizing their effect on the global model [1]. Intuitively, even if their local weights are encrypted, an adversary can leverage the openness to obtain the number of local models sent and do their estimation.

While the benign clients compute their model weights and upload them to the blockchain network after each iteration, the malicious client(s) use a properly crafted boosting factor to launch the scaling attack [48]. The crafted boosting factor depends on the number of participating clients, which an attacker can access due to the openness of the blockchain. The local model of the malicious client ($w_{adv}^{t}$) in such a case is computed as

(3) $$w_{adv_{i}}^{t}=\beta \left(w_{adv_{i}}^{t-1}-\eta \nabla f_{i}\left(W_{g}^{t-1};x_{i},y_{i}\right)\right)$$

where β = $\frac{n}{\eta }$ is a boosting factor that depends on the number of participating clients (*n*) and the learning rate (η). Assuming that we have *v* number of malicious clients and *n* total number of participating clients, the global update in Equation (2) can be rewritten as

(4) $$W_{g}^{t}=\sum_{i=1}^{v}\frac{d_{i}}{D}w_{adv_{i}}^{t}+\sum_{j=1}^{n-v}\frac{d_{j}}{D}w_{j}^{t}$$

Substituting Equations (1) and (3) in (4) will result in a global model update expressed as

(5) $$\begin{array}{c} W_{g}^{t}=\sum_{i=1}^{v}\frac{d_{i}}{D}\beta \left(w_{adv_{i}}^{t-1}-\eta \nabla f_{i}\left(W_{g}^{t-1};x_{i},y_{i}\right)\right)+\\ \sum_{j=1}^{n-v}\frac{d_{j}}{D}\left(w_{j}^{t-1}-\eta \nabla f_{j}\left(W_{g}^{t-1};x_{j},y_{j}\right)\right) \end{array}$$

The loss functions $f_{i}$ and $f_{j}$ in both terms tend to zero as the global model converges when *t* becomes very large. Thus, Equation (5) can be rewritten as

(6) $$W_{g}^{t}=\sum_{i=1}^{v}\frac{d_{i}}{D}\beta \left(w_{adv_{i}}^{t-1}\right)+\sum_{j=1}^{n-v}\frac{d_{j}}{D}w_{j}^{t-1}$$

We rewrite Equation (6) as follows:

(7) $$W_{t}^{g}=\frac{1}{D}\left(\sum_{i=1}^{v}d_{i}\;\beta w_{advi}^{t-1}+\sum_{j=1}^{n-v}d_{j}\;w_{j}^{t-1}\right).$$

We approximate by discarding the second summation, and we obtain

(8) $${\tilde{W}}_{t}^{g}=\frac{1}{D}\sum_{i=1}^{v}d_{i}\;\beta w_{advi}^{t-1}.$$

The approximation error, $E_{t}$, is

(9) $$E_{t}=W_{t}^{g}-{\tilde{W}}_{t}^{g}=\frac{1}{D}\sum_{j=1}^{n-v}d_{j}\;w_{j}^{t-1}.$$

Since $\sum_{j=1}^{n-v}d_{j}\leq D$, we obtain the bound:

(10) $$∥E_{t}∥\leq \frac{1}{D}\cdot \left(D-\sum_{i=1}^{v}d_{i}\right)\;\max_{j}∥w_{j}^{t-1}∥.$$

Thus, the error depends only on the proportion of benign clients and their weight magnitudes. As β grows large, the malicious term scales linearly with β, while the error remains bounded above, so the relative error goes to zero:

(11) $$\frac{∥E_{t}∥}{∥{\tilde{W}}_{t}^{g}∥}\to 0\;\mathrm{as}\;\beta \to \infty .$$

Therefore, the malicious client(s) successfully compromise the global model updates at this iteration *t*, and the global model can be rewritten as

(12) $$W_{g}^{t}\approx \sum_{i=1}^{v}\frac{d_{i}}{D}\beta \left(w_{adv_{i}}^{t-1}\right)$$

All clients download this poisoned global update afterward. This results in the degradation of the overall accuracy of the global model.

### 5.3. FedECPA Defense Algorithm

**Figure 3:**
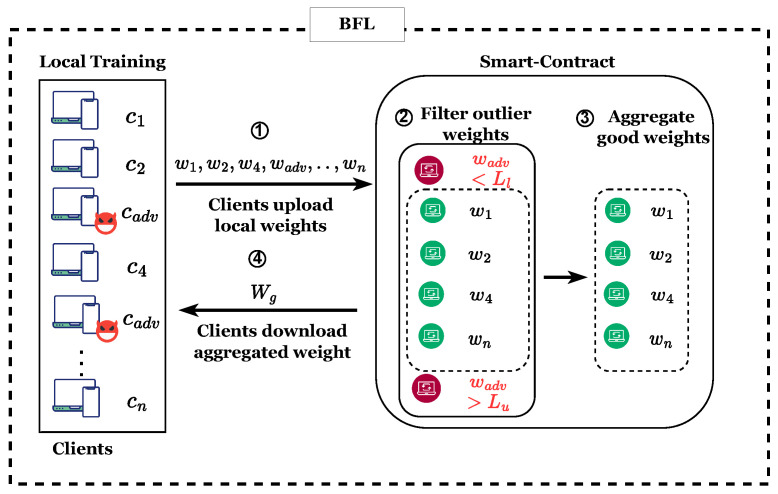
The Architecture of FedECPA. In (1), the participating clients perform local training and upload their weights to the blockchain. (2) These weights are then filtered to exclude the malicious clients’ model weights. (3) The non-malicious clients’ weights are aggregated to generate the global model weight. (4) The global model weight is then downloaded by all clients for the next training iteration.

To defend against scaling attacks, blockchain nodes in our proposed defense mechanism, as explained in Algorithm 1, filter out malicious clients that submit outlier weights before they do FedAvg aggregation. To do so, we employ the interquartile range (IQR) rule as a robust statistical measure that captures the spread of the central 50% of the data while minimizing the influence of extreme values. It is particularly effective for detecting outliers in model weights. In BFL, this technique shows the variation in the clients’ model weights and identifies outlier weights that fall below a lower limit or above an upper limit. These lower and upper bounds depend on the 25th and 75th percentiles, respectively [51]. It should be noted that the global model will still converge while excluding these outlier weights submitted by malicious clients from aggregation. Figure 3 illustrates the high-level overview of FedECPA. We explain the steps involved in the defense in detail as follows.

- *Step 1*: Each participating client performs its training and uploads local (malicious and non-malicious) model weights $\left(w_{1},w_{2},w_{4},w_{adv},\ldots ,w_{n}\right)$ to the blockchain for aggregation (see lines 5–10 in Algorithm 1). It should be noted that the uploaded local weights come from both benign clients $\left(w_{1},w_{2},w_{4},\ldots ,w_{n}\right)$ and malicious clients $\left(w_{adv}\right)$. They are added to the system as transactions.

**Algorithm 1:** Pseudocode of FedECPA 
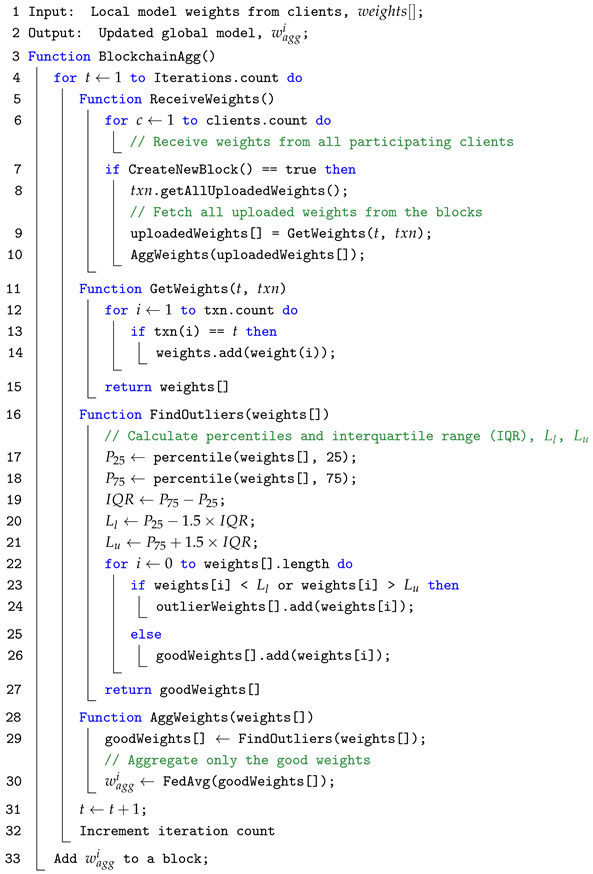

- *Step 2*: After the clients upload local weights, the findOutliers function in the smart contract is invoked by blockchain nodes before performing the aggregations (see lines 16 in Algorithm 1). This function employs the IQR rule to identify malicious clients with outlier weights that exceed a lower limit $L_{l}$ and upper limit $L_{u}$. Note that, following the common practice in some FL-related papers (e.g., [43,52]) which use neural networks for building local models, the notation [x] denotes a two-dimensional vector of variable *x* that is generally adopted in this section. For example, $\left[w_{i}\right]$ in (13) is a two-dimensional vector representing the weights of the local model sent by *i*. These local weights are sorted across the vector dimension of each client. Then, the 25th and 75th percentiles are computed as follows:
  (13) $$\left[P_{25}\right]=\frac{25({\left[w_{v_{i}}\right]}_{n}+1)}{100},\left[P_{75}\right]=\frac{75({\left[w_{v_{i}}\right]}_{n}+1)}{100}$$
  Following that, the IQR is computed as
  (14) $$\left[IQR\right]=\left[P_{75}\right]-\left[P_{25}\right]$$
  Then, the Tukey fence lower and upper limits are computed as
  (15) $$\left[L_{l}\right]=\left[P_{25}\right]-1.5\left[IQR\right]$$
  (16) $$\left[L_{u}\right]=\left[P_{75}\right]+1.5\left[IQR\right]$$
  Suppose any client’s weights fall below $\left[L_{l}\right]$ or above $\left[L_{u}\right]$. In that case, the client is treated as a malicious client, and its local model weights are excluded from the weights aggregation for that particular learning iteration as depicted in Figure 3.
- *Step 3*: Next, the weights returned from the findOutliers function are aggregated to compute the new global model (see lines 28–30 in Algorithm 1).
- *Step 4*: Lastly, the new global model is added to a blockchain and downloaded by clients for the next training iteration (see lines 32 in Algorithm 1).

With our defense mechanism, the outlier model weights from the malicious client(s) are detected and excluded from the overall model aggregation. It should be noted that the benign clients’ weights, even in a non-IID setting, as verified in our experimental results, will not be excluded since they remain within the bounds of $L_{l}$ and $L_{u}$. In contrast, when a substantial boosting factor significantly impacts the global model, the weights of malicious clients will deviate beyond $L_{l}$ or $L_{u}$, leading to their detection and subsequent exclusion, as will be seen in Section 6. *Hence, our approach effectively protects BFL from the scaling attack.*

### 5.4. BFL Implementation

To verify that our proposed defense mechanism works in a blockchain setting, we implemented a BFL smart contract using Solidity on top of the Ethereum public blockchain platform [53]. Smart contracts are deployed and tested locally on Ganache using Truffle [54]. Although the Ethereum virtual machine does not support floating-point numbers and multi-dimensional arrays at the time of this research, the model weights were stored using 256-bit integers and represented as flattened arrays to enable operations on the model weights in Solidity.

The following smart contract functions are implemented for BFL and our defense mechanism.

- GlobalModelRequest: At the beginning of the task, an initial global model is randomly computed in the blockchain. This happens after a requester submits a BFL task request to the blockchain.
- DownloadGlobalWeights: Each client invokes this function to obtain the current global weight. This global weight could be the initial or subsequent global weight.
- UploadWeights: This function is called by clients when submitting their local model weights, $w_{i}^{t}$, to the blockchain network. Once verified, each weight gets recorded as a transaction in a block on the blockchain.
- AggWeights: After the required minimum number of weights from the participating clients have been uploaded to the blockchain network, this function is invoked automatically to compute the aggregation of all the weights. The aggregated weight, $W_{g}^{t}$, is the current global weight for the next training iteration *t*.
- FindOutliers: This function helps to filter out and isolate the outlier weights, $w_{adv}^{t}$, from being included in the aggregation process. To filter the outlier weights, the local model weights must be sorted. In our experiment, the weights were sorted before being uploaded to the blockchain because sorting on the blockchain requires a large amount of gas to be executed. With this function, only weights from non-malicious clients are considered for aggregation in each communication iteration.

## 6. Experimental Setup and Performance Evaluation

This section discusses the experimental setup of this work, the performance evaluation of BFL and FedECPA, and the complexity and security analysis of FedECPA.

### 6.1. Experimental Setup

**Data Setting:**

The MNIST [55] dataset and the CIFAR-10 [56] dataset are used for evaluation in our experiments. The MNIST dataset consists of 60,000 training and 10,000 handwritten testing digits with ten classes. Each sample is a 28-by-28-pixel grayscale image. For the IID setting, the training data samples are shuffled and partitioned equally among 100 clients, with each client receiving 600 training samples that include all classes. For the non-IID setting, the training data samples are partitioned so that each client has access to only two classes [57]. The 10,000 testing samples are used to evaluate the performance of the global model. Similarly, the CIFAR-10 dataset consists of 50,000 training images and 10,000 testing images across ten classes. Each sample is a 32-by-32-pixel color image with three RGB channels. We adopt the same IID and non-IID data partitioning strategies as described for the MNIST dataset.

**Model Setting:** For MNIST, the model setup at each local client was a three-layer deep neural network model with 784 input nodes, 128 hidden nodes, and 256 hidden nodes using the Rectified Linear Unit (RELU), and 10 output nodes using Softmax activation was trained. Adopting a more complex or pre-trained model, such as ResNet, would yield similar training results [58]. Thus, 136,074 parameters were trained using the Adam optimizer, sparse categorical cross-entropy loss, and a learning rate of 0.01. Each client’s model is trained using a batch size of 32 and for 50 epochs. The training is carried out in 100 iterations for the FL task.

For CIFAR-10, the model setup at each local client was a convolutional neural network (CNN) consisting of two convolutional layers and two max-pooling layers. The first convolutional layer has 32 filters with a kernel size of 3 × 3 using the Rectified Linear Unit (ReLU) activation, followed by a 2 × 2 max-pooling layer. The second convolutional layer has 64 filters with a kernel size of 3 × 3 and ReLU activation, also followed by a 2 × 2 max-pooling layer. The feature maps are then flattened, passed through a dropout layer with a rate of 0.5, and finally connected to a fully connected dense layer of 10 output nodes with Softmax activation for classification. In total, 119,658 parameters were trained using the Adam optimizer, sparse categorical cross-entropy loss, and a learning rate of 0.01. Each client’s model is trained with a batch size of 32 for 50 epochs. The risk of overfitting during training is mitigated by the use of mini-batches, a moderate-sized model, and aggregation across multiple clients, which naturally improves generalization in the federated setting.

**FL setup:** To implement the FL part, we used the Flower framework [59], running on an NVIDIA GeForce RTX 3090, 40 GB GPU with a compute capability of 8.6. We simulate a server and 100 clients with the Flower framework, which was implemented with Python 3.10. Each client implemented real-world deep neural network training using TensorFlow-Keras [60] and the above settings. This work assumes that all 100 clients participate in a given learning iteration. After each client’s training, they generate the local model weights and upload them to the server for aggregation.

**Baselines:** The following are the baselines used to evaluate the performance of our proposed defense mechanism:

- *FedAvg* [44]: The traditional FL aggregation technique as expressed in Equation (2).
- *FedAvg with attack*: The traditional FedAvg techniques under scaling attack.
- *Multikrum* [43]: An FL aggregation algorithm that selects clients with the best scores for the local model weights. Specifically, it selects the m∈{1,…,n} weights $W_{1},\ldots ,W_{m}$, with the best scores and aggregates them as $\frac{1}{m}\sum_{i}W_{i}^{*}$. This algorithm is widely adopted as a poisoning attack defense algorithm.

**Performance Metrics:** The performance metrics used in this work are

- *Accuracy:* It measures the efficacy of an ML model by estimating the ratio of accurate predictions of the model to the total number of predictions. In this work, we measure the BFL model accuracy without the attack, the BFL model accuracy with the attack, and the BFL model accuracy with the attack and the proposed defense. Note that accuracy here refers to test accuracy, i.e., the 10,000 testing samples that are assumed to be held aside for validation.
- *Attack Impact Rate (AIR):* AIR measures the effect of the model poisoning attack on the generated model performance. This is measured in terms of the accuracy difference between a general setting without an attack and under attack.
- *Gas Cost:* This is the total cost of gas used for all the operations carried out in the blockchain network. It is calculated by finding the product of the gas used and the unit gas price specified in the transaction: gasUsed * gasPrice. The unit gasPrice in GWEI used for this experiment is 20, and the Gas Cost is paid in Ether, a cryptocurrency.

### 6.2. Performance Evaluation of BFL

**Figure 4:**
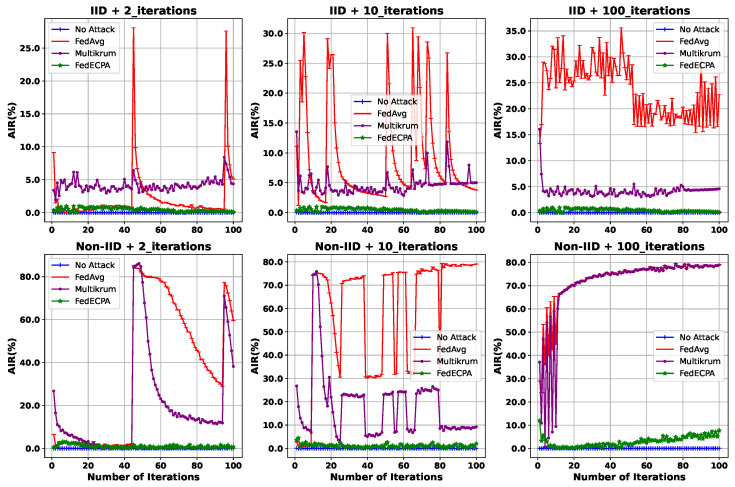
Attack impact rate on BFL for attack strategy in both IID and Non-IID settings. The attack is launched at 2 iterations, 10 iterations, and 100 iterations.

**Impact of the attack frequency on BFL:** Using different attack frequency settings under both IID and non-IID settings, we evaluate the impact of the scaling attack with 100 participating clients, including 10 malicious clients. Figure 4 depicts the AIR when the attack is launched at 2 iterations (45th and 95th), 10 iterations (every 10 iterations), and all 100 iterations. The AIR usually increases at the attack iterations and decreases at non-attack iterations when the model accuracy tries to recover from the attack’s impact. The increase is particularly noticeable when the attack occurs in 2 and 10 iterations. It is observed that the model accuracy drastically drops regardless of the attack frequency employed by the malicious clients. Consequently, AIR for FedAvg is high under the IID setting without any defense mechanism. The AIR for Multikrum is seen to have a value of about 3%, which means that the attack still affects the global model. Nevertheless, FedECPA mitigated the scaling attack effectively with an AIR of 0%, thereby maintaining a high model accuracy in the BFL system. Notably, the AIR for both FedAvg and Multikrum under non-IID settings is significantly high, particularly when attacks persist throughout all training iterations, as they could not resist the scaling attack. However, with FedECPA, the scaling attack had a minimal effect on the global model with a low AIR of about 5%. In subsequent evaluations, we concentrate our analysis on implementing attacks in 2 iterations: t=45 and t=95.

**Figure 5:**
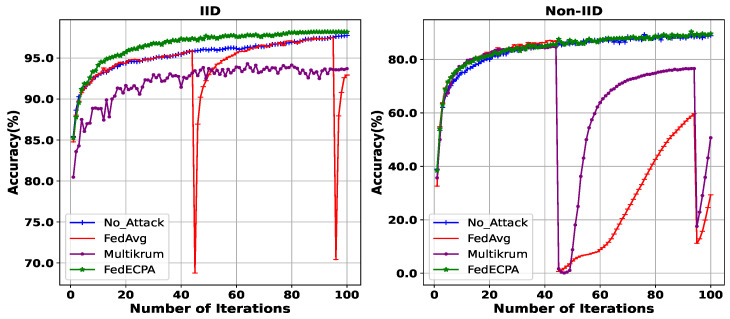
Accuracy for BFL without attack, with an attack on FedAvg, Multikrum, and FedECPA in both IID and Non-IID settings on the MNIST dataset. n=100, m=10%, *t* = 45th and 95th.

**Evaluation of BFL with and without attack (MNIST Dataset):** For our next evaluation, we deployed the scaling attack at iterations 45th and 95th, with 10% of the clients being malicious. The aim is to demonstrate how the model recovers after the attack and if it can fully recover. Thus, we want to show the effect on the global accuracy at the end of the training task and as training progresses after attacks. The results are depicted in Figure 5, where the performance of BFL with the attack FedAvg, without the attack no_attack, Multikrum, and with our proposed defense, FedECPA, is shown. At the 45th and the 95th iterations, where the attack is launched, the accuracy for FedAvg drops to 68% and 70%, respectively. Although a recovery phase becomes apparent during the last communication iterations, the global accuracy at the end of the task is degraded. However, under non-IID settings, both FedAvg and Multikrum demonstrated persistent vulnerability to the attack without recovery, meaning that their global accuracy remains degraded from the effect of the attack.

Similarly, as represented in Table 2 and Table 3, the overall accuracy of the global model for FedAvg at the 100th iteration reduces from 97% to about 93% accuracy in the IID setting and from 89.91% to 29.3% accuracy in the Non-IID setting. MultiKrum maintains relatively low accuracy throughout the training task under IID and has a degraded global accuracy of 50% under Non-IID. In contrast, FedECPA effectively defended against the scaling attack, maintaining a consistent and high global model accuracy under both IID and non-IID settings. This is because the outlier model weights are excluded from the aggregation in FedECPA, resulting in a robust defense against scaling poisoning attacks.

**Figure 6:**
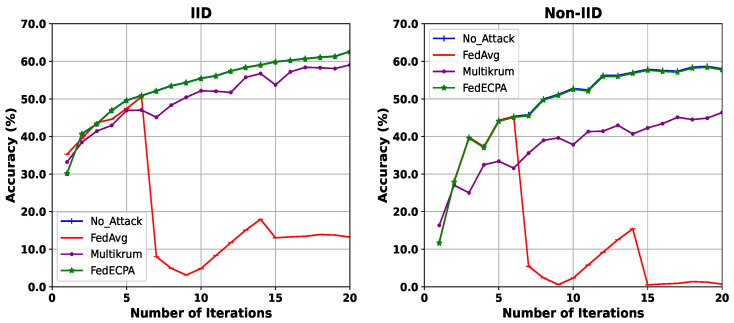
Accuracy for BFL without attack, with an attack on FedAvg, Multikrum, and FedECPA in both IID and Non-IID settings on the CIFAR-10 dataset. n=20, m=10%, *t* = 7th and 15th.

**Evaluation of BFL with and without attack (CIFAR-10 dataset):** For this evaluation, we compared the robustness of different aggregation strategies under IID and non-IID settings, using the CIFAR-10 dataset. With 100 clients and 10% of malicious clients, we deployed the scaling attack at iterations 7th and 15th, with 10% of the clients being malicious. Under IID settings, FedAvg suffers significant degradation when malicious clients launch scaling attacks, as shown in Figure 6. Its accuracy collapses sharply, dropping to 5.4% at the 7th iteration and to 13.02% by the 20th iteration. Although Multikrum maintains moderate robustness with an accuracy of around 58% at the final iteration, it lags behind the no-attack baseline (62.0%) and demonstrates vulnerability during the attack rounds. By contrast, FedECPA closely aligns with the no-attack baseline throughout, reaching 61.3% at the final iteration, thus showing strong recovery and resilience against adversarial interference. Under non-IID settings, the vulnerability of FedAvg becomes even more apparent, as it fails to recover after attack points and maintains degraded accuracy. Similarly, Multikrum only partially mitigates the attack, plateauing below the no-attack performance. FedECPA, however, consistently preserves high accuracy, nearly matching the no-attack baseline across all communication rounds, thereby demonstrating that it is robust even under the more challenging heterogeneous data scenario.

**Figure 7:**
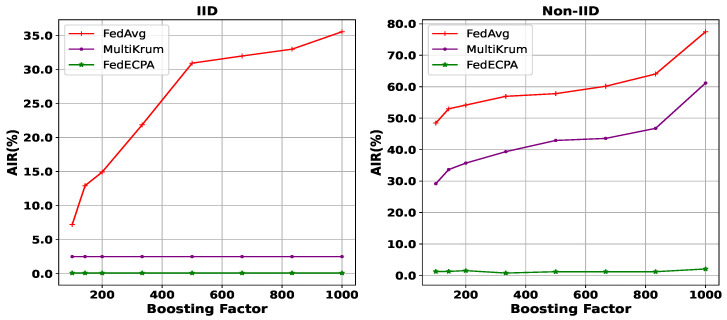
Attack impact rate for varied boosting factors in both IID and Non-IID settings. n=100, m=10%, *t* = 45th.

**Impact of Learning Rate and Boosting Factor:** Next, we evaluate the effect of the scaling attack on BFL under different learning rates and their corresponding boosting factors. In particular, the boosting factors (1000, 833.3, 666.6, 500, 333.3, 200, 142.8, 100) correspond to learning rates (0.01, 0.012, 0.015, 0.02, 0.03, 0.05, 0.07, 0.1), respectively. This mapping highlights the direct relationship between the learning rate and the attack strength: smaller learning rates lead to higher boosting factors, which in turn amplify the effect of the scaling attack. As illustrated in Figure 7, AIR tends to increase with a higher boosting factor (i.e., smaller learning rate), because the scaling multiplier makes the malicious weights dominate the aggregation process. For example, when using the Multikrum technique under the IID setting, the AIR is about 3% with benign settings. However, it rises from 30% to 60% under the Non-IID setting. This indicates the significant influence of scaling attacks on the global model. Thus, FedECPA remains robust across all considered learning rates. Even at the smallest learning rate (η=0.01, corresponding to β=1000), where the scaling attack is most severe, FedECPA maintained an AIR close to 0%. This shows that FedECPA generalizes well across a wide spectrum of practical learning rates.

**Figure 8:**
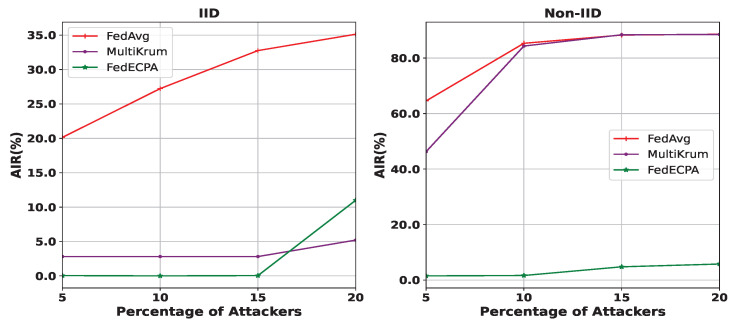
Attack impact rate with increasing percentage of malicious clients in both IID and Non-IID settings. m∈[5%,10%,15%,20%].

**Impact of the percentage of malicious clients: **Figure 8 illustrates the impact of the percentage of malicious clients on BFL. In this experiment, the number of participating clients is fixed to 100 (n=100), while the percentage of malicious clients increases from 5% to 20%. As can be seen, the attack impact rate increased significantly as we increased the percentage of malicious clients. This is due to the increase in the number of malicious local weights (uploaded from malicious clients) and the fact that the first term in Equation (6) will extremely dominate the resulting global model. Compared to Multikrum, FedECPA better resists the scaling attack and maintains a low AIR under the IID setting as long as the percentage of malicious clients was less than 20%, which meets our prior assumption on the percentage of malicious clients. However, under the non-IID setting, FedAvg and Multikrum incurred a high AIR, while FedECPA significantly resisted the attack, maintaining a low AIR. As the percentage of malicious clients increases, FedECPA struggles to fully resist the scaling attack. This is because the malicious clients’ weights no longer appear as outliers, causing the defense mechanism to fail in excluding them from aggregation. However, under the IID setting, the AIR with Multikrum remains lower by approximately 5% when the percentage of malicious clients exceeds 15%. It should be noted that achieving more than 20% of malicious clients is practically difficult or even impossible in FL as the number of clients increases.

**Figure 9:**
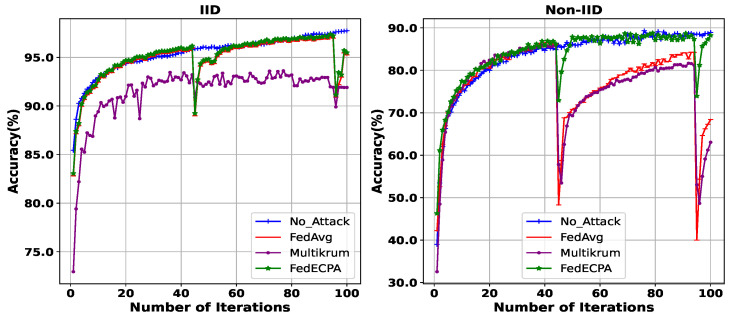
BFL accuracy with the malicious clients’ knowledge of the defense mechanism for FL without attack; No_Attack, with attack; FedAvg, MultiKrum, and FedECPA in both IID and Non-IID settings.

**Impact of the knowledge of malicious clients:** Due to the open nature of the blockchain, which enables participants to access the distributed ledger, the malicious client(s) have access to all the weights submitted by all other participating clients. If, after launching the attack, the aggregated global model is still evaluated with good accuracy, the adversary evaluates the global model weight and deduces that some of the local model weights are excluded from aggregation. With this new information, the adversary knows the defense mechanism. To still achieve the goal of poisoning the global model, it computes and uploads local weights that, although poisoned, fall within the range of weights uploaded by the other benign participating clients. In Figure 9, under the IID setting, we can see that the accuracy for FedAvg, Multikrum, and FedECPA at the iterations when the attack was launched appear to be almost the same because the attacker’s new approach proves to be more challenging to defend against by the blockchain. Nevertheless, it can be seen that the effect of the failure is minimal as the system still maintained a model accuracy of about 90%. However, under non-IID settings, there seems to be better resistance to the attack with FedECPA than the other baselines.

### 6.3. Blockchain Performance Evaluation

**Figure 10:**
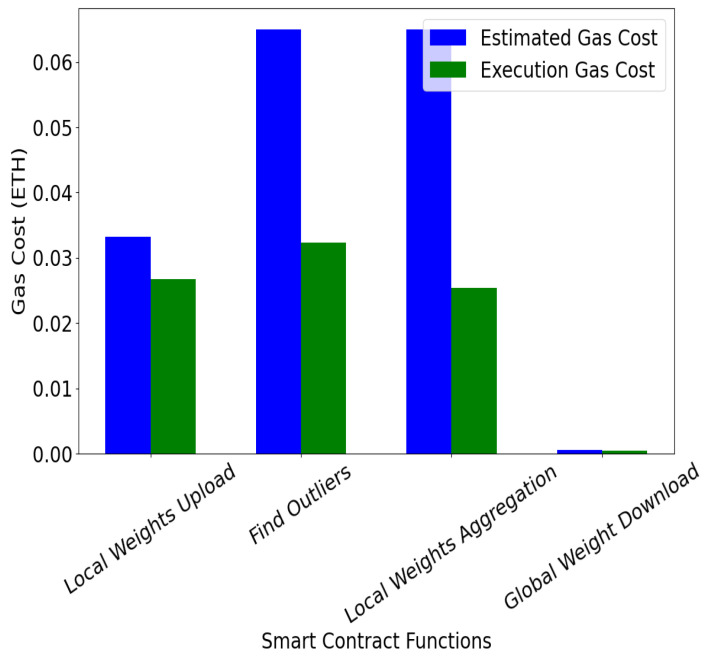
Gas cost for smart contract functions.

**Gas cost for smart contract functions:** To evaluate the blockchain performance of FedECPA, we implemented the smart contracts on a local Ethereum test network using Ganache. The experiments were conducted with 20 blockchain nodes and a block gas limit of 6,721,975. The client nodes interacted with the blockchain by uploading local model weights, triggering the *findOutliers()* and *aggWeights()* functions, and downloading the global model. Gas consumption was measured using the built-in Ganache profiler. In this experiment, we query the gas cost for each smart contract function call by a client or a blockchain node. The Gas Cost is typically the gas used to complete each transaction’s computational and storage requirements in the blockchain network. Figure 10 shows the gas costs of the uploadWeights(), findOutliers(), aggWeights(), and downloadGlobalWeight() smart contract functions. It can be seen that all functions were executed successfully within the gas cost limit for each of the transactions. For example, the estimated gas limit for the aggWeights() and findOutliers() functions was 6500 K, and the execution gas used was 2500 K and 3220 K, respectively, corresponding to approximately 0.00025 ETH and 0.000322 ETH. Also, a 22% increase in the gas consumption is observed for findOutliers() functions. However, the function is still executed without exceeding the gas limit, *proving that the defense can be implemented within a blockchain system.*

**Figure 11:**
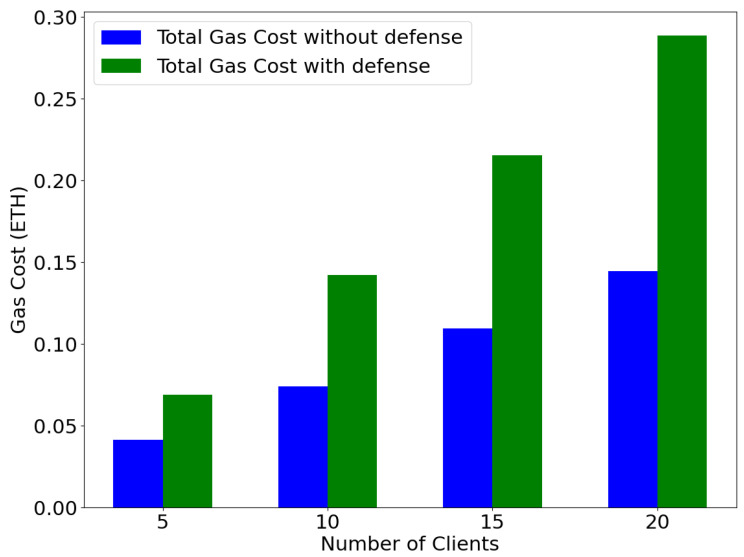
Gas cost based on the increasing number of participating clients, n∈[5,10,15,20].

**Gas cost based on the number of participating clients:** Additionally, we query the total gas cost required based on the number of participating clients with and without the defense mechanism. In this setting, we steadily increase the number of participating clients from 5 to 20. The gas cost steadily increases in both computation cases with and without the proposed defense, as observed in Figure 11. In essence, a gas cost overhead occurs with almost twice the total execution gas cost required by the defense mechanism. This is because, as the number of clients increases, the findOutliers() smart contract function becomes more computationally intensive and requires more gas consumption for a successful execution. Nevertheless, our proposed defense successfully defended against the scaling attack by excluding malicious clients without exceeding the gas limit.

### 6.4. Complexity Analysis of FedECPA

To detect malicious clients with outlier local model weights, the server needs to calculate the 25th and 75th percentiles ($P_{25},P_{75}$), the [IQR], and the Tukey bounds $\left[L_{l}\right]$ and $\left[L_{u}\right]$. This first requires storing all local model weights submitted by the clients in each iteration, which has a space complexity of O(n), where *n* is the number of clients. Calculating the percentiles requires sorting the weights in each iteration, which has complexity O(nlogn). Computing [IQR], $\left[L_{l}\right]$, and $\left[L_{u}\right]$ using Equations (14)–(16) involves only basic arithmetic, while comparing each client’s weight against these bounds to identify outliers has complexity O(n). Since sorting dominates the per-iteration cost, the total per-iteration complexity is O(nlogn). Across *T* iterations, the overall complexity of FedECPA is O(T·nlogn). This demonstrates that the complexity scales linearly with the number of clients per iteration and is practical given available computational resources.

### 6.5. Security Analysis of FedECPA

In Section 4, we put forward some desired security goals that we want to achieve. We show that these goals have been achieved as follows:

**Preposition** **1.** *BFL ensures that the global model is not poisoned.*

**Lemma** **1.** *Deterministic threshold for scaling attack. Let the empirical 25% and 75% percentiles and Tukey fences at a fixed coordinate j be as in *(13)*, *(14)*, *(15)*, and *(16)*:*

$$\left[P_{25}\right],\;\left[P_{75}\right],\;\left[IQR\right]=\left[P_{75}\right]-\left[P_{25}\right],$$

$$\left[L_{l}\right]=\left[P_{25}\right]-1.5\left[IQR\right],\;\left[L_{u}\right]=\left[P_{75}\right]+1.5\left[IQR\right].$$

*Suppose an adversary reports*

$$x_{\mathrm{mal},j}=\alpha m_{j},$$

*with $m_{j}\in R\setminus \left\{0\right\}$. Then,*

(17) $$x_{\mathrm{mal},j}>\left[L_{u}\right]\Leftrightarrow \alpha m_{j}>\left[L_{u}\right]\;\Rightarrow \;\alpha \;>\;\frac{\left[L_{u}\right]}{m_{j}}\;\left(if\;m_{j}>0\right),$$

*and similarly for the lower fence,*

(18) $$x_{\mathrm{mal},j}<\left[L_{l}\right]\Leftrightarrow \alpha m_{j}<\left[L_{l}\right]\;\Rightarrow \;\alpha \;<\;\frac{\left[L_{l}\right]}{m_{j}}\;\left(if\;m_{j}>0\right).$$

We give the Dvoretzky–Kiefer–Wolfowitz (DKW)-based finite-sample argument that yields an explicit probability guarantee that a scaling attack exceeding a computable threshold will be outside the Tukey fence.

**Theorem** **1** (Finite-*n* detection using DKW)**.** *Let the benign client values at coordinate j be i.i.d. draws from distribution F with (population) quantile function $Q\left(p\right)=F^{-1}\left(p\right)$ (right-continuous). Let $F_{n}$ be the empirical CDF from n benign samples and let $\left[P_{25}\right],\left[P_{75}\right]$ denote the corresponding empirical 25% and 75% quantiles (as in (8)). Define the empirical Tukey fences by*

$$\left[L_{l}\right]=\left[P_{25}\right]-1.5\left(\left[P_{75}\right]-\left[P_{25}\right]\right),\;\left[L_{u}\right]=\left[P_{75}\right]+1.5\left(\left[P_{75}\right]-\left[P_{25}\right]\right).$$

*We fix a slack parameter; t∈(0,1/4). Then, with probability at least $1-2\exp(-2nt^{2})$, the empirical upper fence satisfies the bound:*

(19) $$\left[L_{u}\right]\;\leq \;Q\left(0.75+t\right)\;+\;1.5\left(Q(0.75+t)-Q(0.25-t)\right).$$

*Consequently, if an adversary performs a scaling attack $x_{\mathrm{mal},j}=\alpha m_{j}$ and chooses α so that*

(20) $$\alpha m_{j}\;>\;Q\left(0.75+t\right)\;+\;1.5\left(Q(0.75+t)-Q(0.25-t)\right)$$

*then, $x_{\mathrm{mal},j}>\left[L_{u}\right]$ (i.e., the malicious coordinate is outside the Tukey upper fence) with probability at least $1-2\exp(-2nt^{2})$. An analogous symmetric statement holds for the lower fence.*

We proceed in three steps: (A) state DKW, (B) invert the Cumulative Distribution Function (CDF) bound to obtain quantile bounds, and (C) propagate the quantile bounds to the Tukey fence.

(A) DKW inequality. The Dvoretzky–Kiefer–Wolfowitz inequality (Massart’s form) states that for any δ>0,

$$\mathrm{Pr}\;\left(\underset{x\in R}{\sup}\left|F_{n}\left(x\right)-F\left(x\right)\right|>\delta \right)\leq 2\exp\left(-2n\delta^{2}\right).$$

Fix t∈(0,1/4) and set δ=t. Then, with probability at least $1-2\exp(-2nt^{2})$, we have the uniform bound:

(21) $$\underset{x}{\sup}\left|F_{n}\left(x\right)-F\left(x\right)\right|\leq t.$$

(B) Quantile inversion. Let Q(p)=inf{x:F(x)≥p} denote the (right-continuous) population quantile and let $Q_{n}\left(p\right)$ denote the empirical quantile (i.e., $Q_{n}\left(p\right)=\left[P_{p}\right]$). Under the event (21), standard inversion arguments imply that for any p∈(t,1−t),

$$Q\left(p-t\right)\;\leq \;Q_{n}\left(p\right)\;\leq \;Q\left(p+t\right).$$

(21) implies $F_{n}\left(Q\left(p-t\right)\right)\leq F\left(Q\left(p-t\right)\right)+t\leq p$; hence, Q(p−t) is a lower bound for the empirical quantile at level *p*. Similarly, $F_{n}\left(Q\left(p+t\right)\right)\geq F\left(Q\left(p+t\right)\right)-t\geq p$; hence, Q(p+t) is an upper bound for the empirical quantile. Thus, $Q\left(p-t\right)\leq Q_{n}\left(p\right)\leq Q\left(p+t\right)$.)

Apply this to p=0.25 and p=0.75: with probability at least $1-2\exp(-2nt^{2})$,

(22) $$Q\left(0.25-t\right)\leq \left[P_{25}\right]\leq Q\left(0.25+t\right),\;Q\left(0.75-t\right)\leq \left[P_{75}\right]\leq Q\left(0.75+t\right).$$

(C) Propagate to Tukey fence. Using the right-hand inequalities in (22), we obtain an upper bound on the empirical upper fence:

$$\begin{array}{cc} \left[L_{u}\right]\;=\;\left[P_{75}\right]+1.5\left(\left[P_{75}\right]-\left[P_{25}\right]\right)\; & \leq Q\left(0.75+t\right)+1.5\left(Q(0.75+t)-Q(0.25-t)\right)\\ \; & =Q(0.75+t)+1.5Q(0.75+t)-1.5Q(0.25-t), \end{array}$$

which is exactly as in (19). Thus, on the event (21), the empirical upper fence cannot exceed the right-hand side of (19).

Therefore, if $\alpha m_{j}$ strictly exceeds that right-hand side as in (20), then on the event (21), we have $\alpha m_{j}>\left[L_{u}\right]$, i.e., $x_{\mathrm{mal},j}$ lies above the empirical upper Tukey fence. Since (21) holds with probability at least $1-2\exp(-2nt^{2})$, we obtain the stated finite-sample detection probability. The lower-fence statement is analogous to using the left-hand inequalities in (22).

**Preposition** **2.** *BFL ensures the authenticity of the global model, i.e, the global model is non-fake.*

**Proof.**  All participating clients can access the shared blockchain ledger and smart contracts. Also, blockchain nodes have the capability to mine and verify transactions. Leveraging these inherent benefits of blockchain systems, it is ensured that clients’ local weights are verified, and clients perform local training only with verified, non-fake global model weights. The proposition holds under the condition that, as long as mining works correctly and no more than 50% of blockchain participants (miners) are malicious, the authenticity of the global model cannot be manipulated. In other words, if a majority collusion occurs (i.e., >50% malicious clients), then, consistent with blockchain security theory, the system itself is compromised, and adversaries can distort or replace the global model. □

**Preposition** **3.** *No single point of failure, and no third party is required.*

**Proof.**  This comes with the decentralized nature of blockchain and, hence, BFL systems. BFL effectively circumvents the risk of a single-point failure that could potentially occur with a centralized server in traditional FL. In addition, it ensures that miners and verifiers are part of the blockchain system; therefore, we do not require any third party. □

## 7. Conclusions

BFL recently evolved to address the challenges of conventional FL systems. Nevertheless, it comes with some vulnerabilities. One of the vulnerabilities is the model poisoning attack due to the blockchain system’s openness. This paper demonstrated how malicious clients successfully poison the global model of a BFL system. Our experiments focused on the scaling attack because of its maximum effect on the global model compared to other common model poisoning attacks. Furthermore, we explored various attack strategies and client configurations, including both IID and non-IID settings, to investigate the impact of these attacks on the global model performance. Subsequently, FedECPA, an efficient countermeasure against scaling-based model poisoning attacks in blockchain-based FL, was proposed to counter the attacks by employing the IQR rule, a statistical approach to detecting and filtering out outliers. The experimental results showed the efficacy of the defense mechanism when the percentage of malicious clients does not exceed 20% of the participating clients and when the malicious client is not honest and non-curious. Compared to the baseline methods, FedECPA achieved an overall accuracy of 98% under the IID setting and 89% under the non-IID setting on the MNIST dataset. Consistent performance was also observed on the CIFAR-10 dataset. In the future, we will extend our experiments to include scenarios of the model poisoning attack by colluding clients. Furthermore, we aim to develop a more robust and computationally efficient defense mechanism against adaptive poisoning attacks in BFL.

## Figures and Tables

**Table 1:** Comparison of related works on Blockchain-FL poisoning defenses.

Property	FedECPA (Ours)	Blade-FL [15]	Krum [27]	Shield-FL [23]	ADFL [24]	FLDetector [25]
Technique/ Approach	IQR-based detection of outlier model weights; exclude outliers before aggregation	Clients are trainers + miners; PoW secures updates	Selects local model most similar to others	Cosine similarity between local and global weights	Clients generate proofs via garbled aggregation	Predicts client updates, assigns consistency score
Strengths	Detects malicious clients every round; robust to a range of attack strategies	Reduces privacy leakage; strong blockchain security	Limits impact of single malicious client	Detects distant outliers	Verifiability; privacy-preserving	Detects sudden deviations
Limitations	Fails if majority are malicious	No malicious client detection	Fails if majority are malicious	Fails if poisoned model is near aggregated weights	High overhead; weak against adaptive adversaries	Misses consistent but poisoned updates

**Table 2:** Performance comparison of algorithms under attack in the IID setting.

Learning Iteration	Accuracy		
	FedAvg	MultiKrum	FedECPA
45th	0.69	0.92	0.97
50th	0.92	0.93	0.98
95th	0.70	0.93	0.98
100th	0.93	0.94	0.98

**Table 3:** Performance comparison of algorithms under attack in Non-IID setting.

Learning Iteration	Accuracy		
	FedAvg	MultiKrum	FedECPA
45th	0.01	0.16	0.87
50th	0.04	0.01	0.87
95th	0.11	0.18	0.89
100th	0.29	0.51	0.89

## Data Availability

The original contributions presented in the study are included in this article. The primary datasets used for the experimental analysis are the MNIST and CIFAR-10 datasets, which have been appropriately cited in the manuscript.

## Glossary

The notations used in this paper are presented in the following summary of notations.

**Notation**	**Description**
$w_{i}$	Local model weights of client *i*
$w_{adv}$	Local model weight of a malicious client/attacker
$W_{g}$	Global model weight
η	Learning rate
*n*	Number of participating clients
*v*	Number of malicious participating clients/attackers
*m*	Percentage of attackers
β	Boosting factor; $\frac{n}{\eta }$
*t*	Iteration/Round
$P_{25}$	25th percentile of the local model weights
$P_{75}$	75th percentile of the local model weights
Acc	Model accuracy
IQR	Inter quartile range of local model weights
$L_{l}$	Lower limit of the IQR
$L_{U}$	Upper limit of the IQR
AIR	Attack impact rate
